# A Review on Electrical Impedance Tomography Spectroscopy

**DOI:** 10.3390/s20185160

**Published:** 2020-09-10

**Authors:** Juliana Padilha Leitzke, Hubert Zangl

**Affiliations:** Institute of Smart Systems Technologies, Sensors and Actuators, Alpen-Adria-Universität Klagenfurt, 9020 Klagenfurt, Austria; hubert.zangl@aau.at

**Keywords:** electrical impedance tomography spectroscopy, multi-frequency electrical impedance tomography, image reconstruction

## Abstract

Electrical Impedance Tomography Spectroscopy (EITS) enables the reconstruction of material distributions inside an object based on the frequency-dependent characteristics of different substances. In this paper, we present a review of EITS focusing on physical principles of the technology, sensor geometries, existing measurement systems, reconstruction algorithms, and image representation methods. In addition, a novel imaging method is proposed which could fill some of the gaps found in the literature. As an example of an application, EITS of ice and water mixtures is used.

## 1. Introduction

The distribution of electrical impedance in a volume varies according to the materials it is composed of since different materials will present distinct electrical properties. Electrical Impedance Tomography (EIT) is the inverse problem where the impedance in a region of interest is determined by measuring currents or voltages at the electrodes located on the boundaries [1] and a review on the topic can be found in [2]. EIT is a combination of Electrical Resistance Tomography (ERT) and Electrical Capacitance Tomography (ECT). In ERT the resistance component (e.g., conductivity) is used and in ECT the capacitance component (e.g., permittivity) [3]. It is important to note that in the literature, EIT is often defined as a method that uses the conductance component, as in [4].

The inverse problem of determining the material distribution given certain boundary measurements is ill-posed, therefore not only one solution might exist and so far many image reconstruction algorithms have been developed to numerically reconstruct material distribution [5,6,7,8]. EIT might not bring the best resolution comparing to other tomography methods, but for many applications, this drawback is compensated by the possibility of portability, low cost and safety of such a system.

EITS, which is also known as Multi-frequency EIT, is created by combining EIT and Electrical Impedance Spectroscopy (EIS), which analyzes the frequency-dependent behavior of materials in the frequency domain [9], into one system which is capable of reconstructing material distributions based on the frequency-dependent characteristics of different substances. This topic was initially developed mainly for the classical circular electrode geometry in medical applications, starting with dual-frequency systems [10,11] and later on evolving to multi-frequency systems [12,13], continuing to be improved for different applications. In [14] not only 2D but also 3D EITS reconstruction was performed by imaging different sections of the human head. More recently, complete three-dimensional reconstructions have been performed [15,16].

The main reason why this method has been mainly developed in the medical field is that the impedance of different biological tissues have characteristic behavior, and through EITS it is possible to image different types of tissues in the human body in a safe and low-cost way. This could allow the development of systems which could be available in remote locations where hospitals are not so easily accessible, or in ambulances where portability is essential. EITS has been used in many studies for medical applications such as imaging the breast and breast cancer detection [15,17,18,19], skin cancer detection [20], brain stroke classification [14,21], changes in lung water content in neonates [22], and more.

EITS has also been a promising technology for other applications, such as for material accretion detection, as proposed in [23] for monitoring of ice and water mixtures. However, challenges such as temperature dependency of the electrical properties and how environmental conditions will affect the structure of the ice can present a challenge [24].

In this paper, we present a review of EITS focusing on physical principles of the technology, sensor geometries, existing measurement systems, reconstruction algorithms, and image representation methods. As an example of application for comparison of different imaging methods we chose ice and water mixtures using a circular electrode geometry. Since reviews can be found in the literature for EIT and EIS, but not EITS, the aim of this article is to present the state-of-the-art from its basic principles in order to familiarize aspiring researchers who would like to further develop the field.

## 2. Physical Principles of EITS

The basic physical foundations of EIT derive from Maxwell’s equations, which can be found in [25,26]. We begin by approximating the electric field E (see Appendix A) as E=−▽ϕ, where ϕ is the electric potential. Considering Ohm’s law J=σE, where J is the current density, E is the electric field and σ is the electrical conductivity, we obtain J=−σ▽ϕ. Assuming charge conservation, from ▽J=0, Kirchoff’s law can then be deduced ▽·σ▽ϕ=0.

From the conservation of current, or Gauss theorem, we have that $\int_{\partial \Omega }^{}j=0$, where ∂Ω is the boundary of the body Ω. Considering the current density at the border j=−J·n=σ▽ϕ·n, where n is normal to the boundary ∂Ω.

The boundary condition on electrode *l* is:

(1) $$\int_{\Omega_{l}}^{}\sigma \frac{\partial \phi }{\partial n}=I_{l}$$

for l=1,2…,L in a system with *L* electrodes. It can also be assumed that away from the electrodes $\frac{\partial \phi }{\partial n}=0$ and that ϕ is constant on electrodes ▽ϕ×n=0.

In EITS, the conductivity can be replaced by its complex format, or the complex admittivity, function of conductivity σ and permittivity ε which are frequency dependent:

(2) γ=σ+iωε

## 3. Electrical Characteristics of Materials

Polarization is the relation between molecular properties and its charges when subjected to the influence of an electric field [27]. The main polarization processes from the higher frequency process up to the lowest frequency process are: atomic or electronic polarization, ionic polarization, dipole or orientation polarization, and space charge polarization [28]. In the electronic polarization, the electric field displaces the electron density of the atom, and in ionic polarization the displacement is of ions. In orientation polarization, there is a rotation of molecular dipoles in the direction of the field. In space charge polarization, free charges move through the dielectric according to the electric field.

The polarization can be related to the electric field *E* by

(3) $$P=\varepsilon_{0}\chi E$$

in which χ is the electric susceptibility related to the relative permittivity $\varepsilon_{r}$ by $\varepsilon_{r}=1+\chi$ and $\varepsilon_{0}$ is the permittivity of vacuum.

EITS makes use of the effects of electronic and orientation polarization *P* to identify material distributions by their permittivity or conductivity.

The polarization process in dielectrics is represented by the model of the complex permittivity ε [29] defined in Equation (4), a model that is suitable for a considerable number of substances, in particular ice.

(4) $$\varepsilon =\varepsilon_{\infty }+\frac{\left(\varepsilon_{s}-\varepsilon_{\infty }\right)}{1+{\left(j\omega \tau_{0}\right)}^{1-k}}$$

where $\varepsilon_{\infty }$ is the permittivity in high frequency, $\varepsilon_{s}$ is the static permittivity, ω is the angular frequency, $\tau_{0}$ is the relaxation time, and *k* is a parameter that can vary between 0 and 1. In the case of ice, which is our application in this paper, this expression could also be written as a Debye relaxation process, where k=0, and defined as

(5) $$\varepsilon =\varepsilon^{\prime }-j\varepsilon^{\prime \prime }$$

(6) $$\varepsilon^{\prime }=\varepsilon_{\infty }+\frac{\left(\varepsilon_{s}-\varepsilon_{\infty }\right)}{1+\omega^{2}\tau_{0}^{2}}$$

(7) $$\varepsilon^{\prime \prime }=\frac{\omega \tau_{0}\left(\varepsilon_{s}-\varepsilon_{\infty }\right)}{1+\omega^{2}\tau_{0}^{2}}$$

Or also in terms of complex conductivity σ as

(8) $$\sigma =\sigma^{\prime }-j\sigma^{\prime \prime }$$

(9) $$\sigma^{\prime }=\sigma_{s}+\frac{\omega^{2}\tau_{0}^{2}\left(\sigma_{\infty }-\sigma_{s}\right)}{1+\omega^{2}\tau_{0}^{2}}$$

(10) $$\sigma^{\prime \prime }=\frac{\omega \tau_{0}\left(\sigma_{\infty }-\sigma_{s}\right)}{1+\omega^{2}\tau_{0}^{2}}$$

where $\sigma^{\prime }$ is the real conductivity, $\sigma^{\prime \prime }$ is the complex conductivity, $\sigma_{s}$ the static conductivity, and $\sigma_{\infty }$ the conductivity at high frequency.

**Figure 1:**
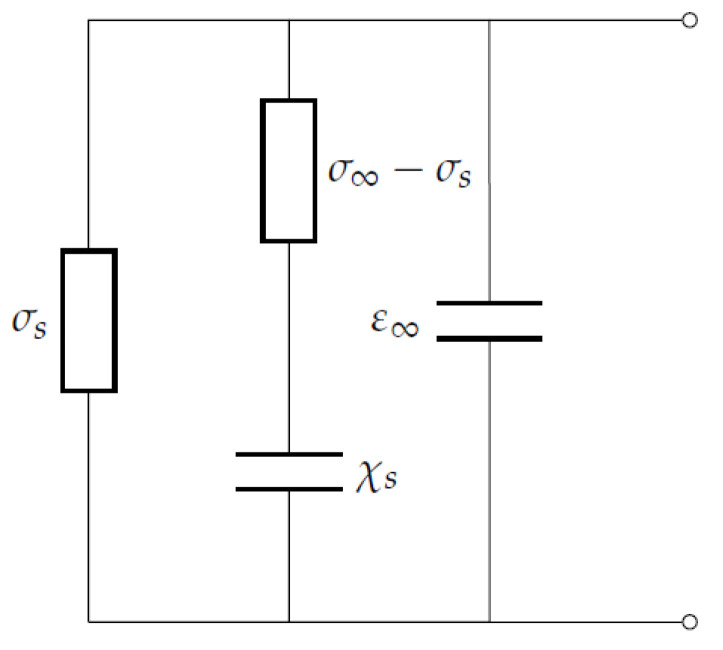
Equivalent circuit of an ice sample considering ideal electrodes.

Ice can be electrically modelled as in Figure 1 [30]. For a plate of thickness *L* and area *A*, we could model it as a capacitance $C=\varepsilon \varepsilon_{0}A/L$ and a conductance G=σA/L, which results in impedance Z=R+iX, where R=1/G and X=1/ωC.

**Figure 2:**
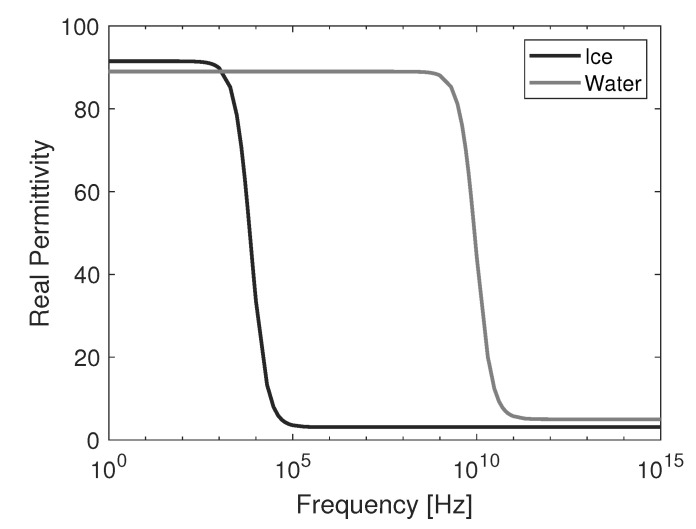
Real permittivity of ice and water.

**Figure 3:**
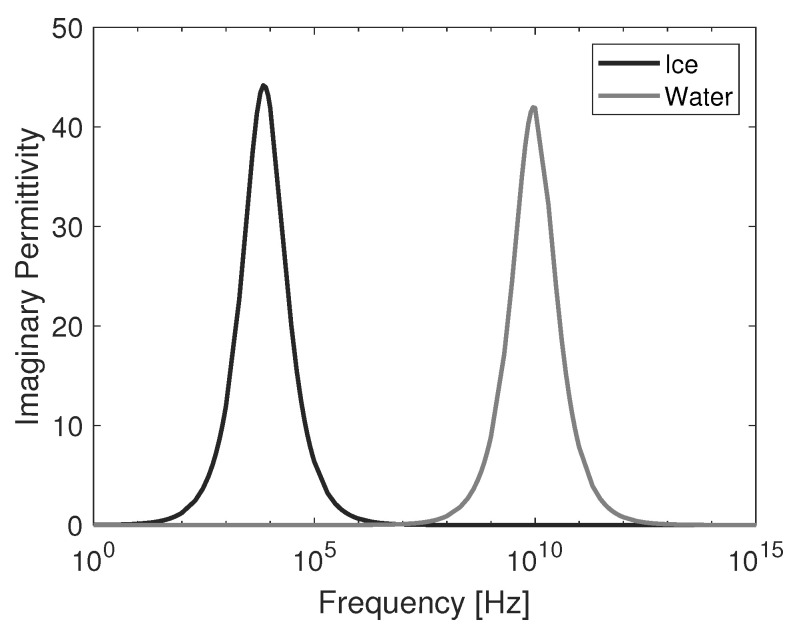
Imaginary permittivity of ice and water.

Based on this model and parameters according to [31,32], Figure 2 and Figure 3 compare the real and imaginary permittivity of pure water and ice at 0 ${}^{\circ }C$ over the frequency range from 1 Hz to 1 PHz. The different relaxation times can be clearly seen on the frequency spectrum.

Many other applications exist in the literature that explore these frequency-dependent electrical properties of materials, especially in biology and medical applications. These properties can be important for portable detection systems for breast cancer [15,17,18,19] since healthy breast tissue has a different frequency behavior as compared to cancerous tissue, as well as brain stroke [14,21], which uses the difference in the behavior of tissue according to the stroke type to better classify it, and even more applications as in [20,22]. In [33], the electrical properties of biological tissues and their frequency dependency is mathematically analyzed in order to show that they are related to the tissue composition and physiology, which could improve modeling in many applications in the medical field, this principle is used in [34,35].

## 4. Sensing Methods

The architecture used for the sensing method can mainly be classified according to the electrode geometry and measurement system technology. Equally important is how noise and errors are addressed during measurement, considering the selected architecture.

### 4.1. Electrode Geometry

In an EITS system, currents or voltages can be applied to the electrodes and voltages or currents can be measured, respectively. The main measurement principles are single-ended, with the signal on the electrode measured with reference to ground, or differential measurement, where the signal is measured between electrodes [25].

**Figure 4:**
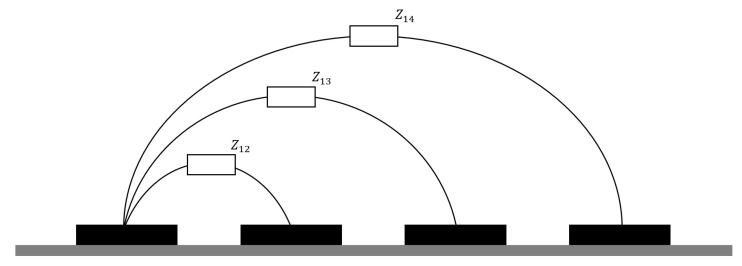
Differential measurement setup example with four electrodes. The equivalent impedances for the first transmitter electrode are shown, these values together with the equivalent impedances obtained with the other transmitter electrodes will generate the measurement matrix.

In Figure 4a simplified differential measurement for a system with four planar electrodes is exemplified. Combination measurements between all electrodes form the measurement matrix which is used in the reconstruction.

#### 4.1.1. Two-Dimensional EITS

**Figure 5:**
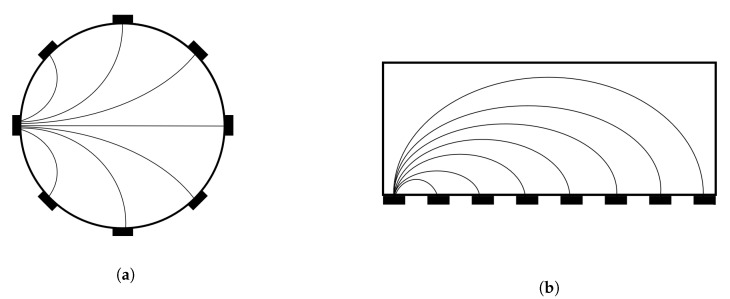
Example of 2D electrode geometries with eight electrodes. (**a**) Traditional circular electrode geometry, (**b**) planar electrode geometry. Field lines are exemplified for one electrode considering differential measurement with respect to the other electrodes. Typically, all combinations between electrodes are evaluated for Electrical Impedance Tomography Spectroscopy (EITS).

The electrodes are traditionally placed around the region to be imaged, as shown in Figure 5a. However, in more recent applications, electrodes have also been placed in a planar geometry which covers only one of the boundaries, as shown in Figure 5b. This can be useful when imaging material accretion or also in robotic applications. Here a cut section is displayed, in which the electrodes are shown in black.

#### 4.1.2. Three-Dimensional EITS

**Figure 6:**
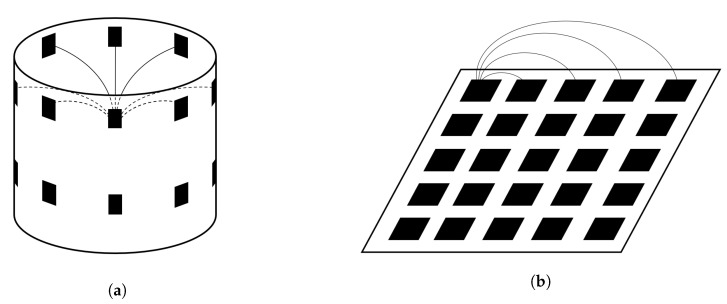
Example of 3D electrode geometries with electrodes shown in black. (**a**) Traditional circular electrode geometry, (**b**) planar electrode geometry. Field lines are exemplified for one electrode and one layer, considering differential measurement. Depending on the evaluation circuitry, the coupling between different layers can also be utilized.

Variations of the electrode setups used for 2D EITS are currently also used for 3D EITS. In Figure 6a an example is shown with two layers of electrodes around a cylindrical shaped object and in Figure 6b a plane of electrodes is exemplified.

In [14], sections of an object are measured around a sphere for frequency difference imaging. This can be particularly useful for some medical applications where many sections can be of interested as currently used for traditional tomography, or for multi-phase flow investigations in industry. In [15] a 3D planar electrode geometry is developed originally for breast cancer detection, but it could also be of interest for other applications.

### 4.2. Measurement Systems

Measurement systems for EITS can be analog or digital, however most recent developments have been based on digital technologies, using Digital Signal Processors (DSP) and Field Programmable Gate Arrays (FPGA), increasing precision and bandwidth when comparing to analog systems [25]. Digital systems also have other advantages, such as higher flexibility and lower complexity. FPGA-based systems have further advantages since they can provide hardware speed and also the possibility of making compact measurement systems.

EITS systems can have a single-source or multiple-source architecture [25]. The former can present the advantage of less hardware, while the latter has fewer issues with stray capacitance since it does not require the use of multiplexers and also a reduced measurement time since measurements can be done in parallel.

The signal generation can be discrete or continuous. In discrete systems, the signal is generated one frequency at a time in sequence, or a few frequencies are added in the same signal and generated simultaneously. However, in continuous systems, a signal is generated which covers the complete frequency spectrum for the operating frequency range. Systems can have different drive patterns, the most common being adjacent and opposite. In adjacent systems, the signal is injected and measured between the nearest electrodes, and in opposite systems, between the pairs of electrodes which are the most distant from one another.

**Figure 7:**
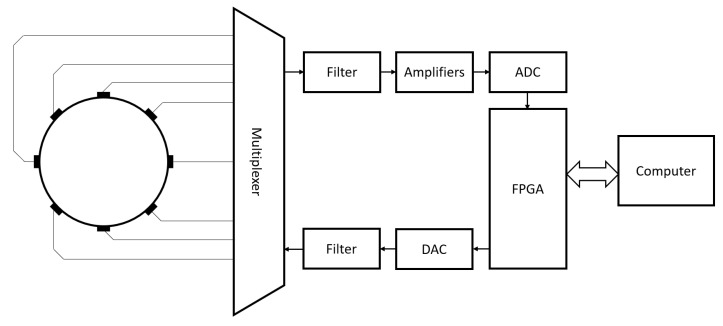
Example of an EITS system consisting of eight electrodes, a single source, and which is digital Field Programmable Gate Arrays (FPGA)-based.

In Figure 7, we show the basic blocks of an EITS system which has eight electrodes, a single source and is digital FPGA-based. The control and reconstruction software are in the computer, while in the FPGA the signal to the transmitter is generated and the receiver data is read. In this example, a multiplexer is used to route the measurement hardware to the electrodes. The receiver signal is generally filtered in a Low-Pass Filter, amplified accordingly with a fixed gain amplifier, which should have low noise and high Common-Mode Rejection Ratio (CMRR), or a programmable gain amplifier, and converted in an Analog-to-Digital Converter before being read in the FPGA. As for the transmitter signal, it might require a Digital-to-Analog Converter and an additional Low-Pass Filter for a smoother signal.

A well-known EITS system is the UCLH Mk 2.5 [36], based on its former version, the UCLH Mk 2 [14], and also on the Sheffield Mk 3.5 [37], which has a Signal-to-Noise Ratio (SNR) of 40 dB with a frequency range from 2 kHz to 1.6 MHz. The frequency range of the UCLH Mk 2.5 goes from 20 Hz to 256 kHz and it incorporates the multifrequency measurement property of the Sheffield Mk 3.5, however it uses the single-source architecture of the UCLH Mk 2 for more flexible electrode configuration.

The OXBACT-3 [38] was one of the first real time DSP systems for EITS. A more recent version, the OXBACT-5 system [39] introduced in 2008 is FPGA-based.

In [18], a system was developed for multilevel measurement of the breast, which was able to do 3D reconstruction. The system consisted of 64 chanels and it was DSP/FPGA based. The frequency ranged from 10 kHz to 10 MHz and it had 65 dB SNR in the worst case for the higher frequency.

The system developed by the Kyung Hee University for EITS operates real-time with a frequency range from 10 Hz to 500 kHz. It was developed using a DSP together with FPGA technology. In [40], it was called KHU Mark1, later being improved into the KHU Mark2 in [41] and KHU Mark2.5 [42], which includes automatic calibration. The system is capable of maintaining an SNR of 80 dB and it has configurable electrode configuration without using multiplexers in a multiple-source architecture.

Many commercial impedance analyzers in the market offer enough accuracy to be used in an EITS system as shown by [43] where an impedance analyzer is used together with a multiplexer, however, they can be expensive and bulky.

An FPGA-based system was developed in [44] using a single-source architecture. This system had a single-source architecture, configurable electrode configuration, SNR higher than 90 dB and discrete frequencies varying between 100 Hz and 10 MHz. Other single-source FPGA-based systems were developed by [16,45].

A more recent FPGA-based system called Spectrum-Based Wideband Electrical Impedance Tomography (SWEIT) was developed in [46]. Using a chirp signal covering from 1 kHz to 1.1 MHz and a multisinusoidal signal, it has a broad spectrum range. It has 16 electrodeas and an average SNR is 56 dB, with a worst case of 40 dB.

In Table 1 a comparison between the main digital EITS systems in the literature is presented.

### 4.3. Measurement Noise and Error

Since noise is an important factor to be considered in a measurement system, work has been done in the literature with the focus on investigating sources of noise and error. Stray capacitances, common-mode effects, and contact impedance are known as the main causes of instrumentation errors [47]. In systems relying on multiplexers, input and output capacitances as well as other issues such as ’on’ resistance can cause extra sources of errors [25].

In [48], systematic errors in EITS in frequencies ranging from 10.24 to 81.92 kHz for medical applications are discussed. The main sources of errors are classified as the transimpedance errors, cable movement errors, electrode impedance errors, and random noise and drift. Calibration measurements performed with a frequency-independent material are the usual approach that can reduce such errors, noise and drifts. In this study, since conductivity was the property of interest, a saline phantom was used.

A review of errors of EITS instrumentation is presented in [47], which explains why this method is still not accepted in some applications mainly in medical fields where it has been proven to allow a fast, low-cost and portable diagnoses. It was suggested that an accuracy better than 0.1% will be required over a broad frequency range from 10 Hz to 10 MHz and a broad range of loads to make this technology appropriate for medical use.

A common way of quantifying the noise in EITS instrumentation is the SNR through the operating frequency range. The average SNR at frequency *f* for a certain channel can be defined as

(11) $$SNR_{f}=10\cdot {log}_{10}\left(\frac{\sum_{n=1}^{N}V_{n}^{2}}{\sum_{n=1}^{N}{\left(V_{n}-\bar{V}\right)}^{2}}\right)$$

where *N* is the total of repeated measurements, $V_{n}$ is the voltage measurement *n* and $\bar{V}$ is the mean of the voltage measurements, equation which shows the relation between signal power and the power of the noise in dB. The average SNR can be defined as the average value of the SNR considering all the frequencies of operation, measurement samples and channels since this value will vary at different frequencies, instants in time, and measurement channel. For SNR measurement, a material should be used which is not frequency dependent in the operation range in order to maintain the same impedance at all frequencies. Materials which are commonly used are a saline solution or air.

However, there are also other parameters which can be of interest to evaluate an EITS system. In the literature, system accuracy is also used and defined as

(12) $$\left|\frac{\left(Z_{expected}-Z_{measured}\right)}{Z_{expected}}\right|\times 100\%$$

where the measured impedance $Z_{measured}$ is compared to the expected value $Z_{expected}$.

Another way of evaluating a system is the CMRR, which is defined as

(13) $$CMRR=20\times {log}_{10}\left(\frac{V_{diff}}{V_{cm}}\right)$$

where $V_{cm}$ is the commom mode voltage and $V_{diff}$ the differential voltage.

## 5. Image Reconstruction Algorithm

An image reconstruction algorithm will calculate an estimation of the material’s spatial distribution from the measurement data for each respective image pixel in the region of interest. Considering just a single frequency, EITS turns essentially into Electrical Impedance Tomography (EIT). In order for a problem to be well-posed, a solution must exist, be unique, and depend continuously on the data, which is not the case for EIT that has an ill-posed nature [25].

Reconstruction algorithms are usually separated into iterative methods, which are more computationally intensive, and non-iterative methods. They can also be classified as direct, regularization-based, statistical and machine learning reconstruction methods.

Some excellent reviews for EIT reconstruction algorithms were written on this topic in previous years [5,6,7], as well as a more recent work with focus on latest algorithm developments [8]. A review on EIT with a description of the main developed systems and reconstruction algorithms, with focus on clinical applications, can be found in [49], where EITS systems are also addressed. Furthermore, a free MATLAB toolbox called Electrical Impedance Tomography and Diffuse Optical Tomography Reconstruction Software (EIDORS) was developed and is available online for anyone interested in using some of the main reconstruction algorithms for EIT [50].

Below we present an overview of reconstruction algorithms with focus on application in EITS and a brief summary in Table 2.

### 5.1. Main Algorithms Used for EITS

The main algorithms still used for EITS application are direct and regularization-based methods. The regularization-based Singular Value Decomposition (SVD) algorithm, also used as the Generalized Singular Value Decomposition (GSVD) or the Truncated Singular Value Decomposition (tSVD) is widely used for EITS applications, as can be seen in [21,42,51]. This algorithm was introduced by [56] and further developed by [57,58].

Considering a measurement matrix *Y*, sensitivity matrix *a*, image pixels matrix λ and measurement error *e* we consider that

(14) Ya=λ+e

The error *e* which should be minimized can then be defined as

(15) $$min\left\{{∥Ya-\lambda ∥}_{2}\right\}$$

The M×N matrix measurement matrix *Y* can also be represented as

(16) $$Y=U\Sigma V^{T}$$

where *U* is an M×M matrix, *V* is an N×N matrix and Σ is an M×N matrix. The diagonal elements of Σ are the singular values, which are defined as the non-negative square root values of the eigenvalues of $A^{T}A$. It is also defined that $U^{T}U=V^{T}V=I$.

The inverse of *Y* cannot be easily obtained since it does not exist for EIT applications. Therefore, the pseudoinverse of *Y* which is $V\Sigma^{-1}U^{T}$ is used to determine a solution.

Tikhonov Regularization was initially developed in [59] and further studied in [60,61]. In this approach a regularization parameter α is assumed and the solution can be expressed by

(17) $$min\left\{{∥Ya-\lambda ∥}_{2}+\alpha {∥\lambda ∥}_{2}\right\}$$

Generalized Tikhonov Regularization is defined as

(18) $$min\left\{{∥Ya-\lambda ∥}_{2}+\alpha {∥L\lambda ∥}_{2}\right\}$$

where *L* is a regularization matrix, which for the Tikhonov Regularization is defined as *I*.

Tikhonov Regularization is used for EITS in [62] and also in [45] together with an iterative algorithm called Generalized Vector Sampled Pattern Matching (GVSPM), which can converge without the need of an empirical regularization factor.

Regularised Newton Methods, which are iterative, have been used in the literature for EITS image reconstruction. A Newton-Raphson based algorithm was used to perform EITS for breast imaging in [17] together with absolute imaging representation. A Gauss-Newton method is used for EITS in [43].

The D-Bar Method is a direct reconstruction method developed in [52], where absolute imaging was made for complex conductivity. Its theory is based on [63], which shows that a complex valued coefficient with small imaginary part can be recovered from the knowledge of the Dirichlet-to-Neumann map.

The Sheffield Filtered Back-Projection was first developed in [64] and further developed in [65] for EIT. It is used in [48] for EITS investigation of systematic errors.

### 5.2. Recent Developments

Statistical modelling and inference for EIT has been reviewed in [66] and also in [67] with a focus on Markov Chain Monte Carlo. This iterative statistical method could also be applied for EITS.

Optimal Approximation is a non-iterative statistical method. This method has been used for EIT, but not yet used for EITS other than in simulations [23,53]. It is well described in [7] and used in [68] for robotic applications. It is essentially an implementation of a Bayesian Linear Minimum Mean Square Estimation (BLMMSE) approach. The Optimal First Order Approximation (OFOA) is the linearization of $\hat{\varepsilon }=E\left\{\varepsilon |y\right\}$ given by

(19) $$\hat{\varepsilon }=Wy+B$$

(20) $$W=C_{\varepsilon y}C_{yy}^{-1}$$

(21) $$B=\hat{\varepsilon }-W\hat{y}$$

where $C_{yy}$ is the auto-covariance matrix of the measurements, $C_{fy}$ is the cross-covariance matrix between the measurements and the permittivities, $\hat{\varepsilon }$ is the expected value of the permittivity, $\hat{y}$ the expected value of the measurements and *y* the obtained measurements.

In [54], the iterative and regularization-based Group Sparse Reconstruction Algorithm (GIST) was recently developed aiming at EITS applications, but demonstrated only through numerical experiments.

Recently, some studies have been made to apply machine learning to EIT [69,70]. In [55], a reconstruction-classification method is used for EITS, where a label is assigned for each element stating which tissue is present. The drawback of this method is that prior information is needed with high accuracy of the values of the electrical properties.

Studies have also been made in [71] combining the Particle Swarm Optimization method (PSO) for Artificial Neural Networks (ANN) training and EIT. However, no multifrequency analysis was made.

## 6. Image Representation

The reconstructed image can be absolute, but it can also be a differential image, which fuses time and/or frequency reconstructed images into one final image that can better compile the information depending on the application.

In [72] a comparison between the main image representation methods was presented, including time difference, frequency difference, weighted frequency difference and adjacent frequency difference. However, not all imaging methods were covered in the study.

In addition, we would like to present a novel imaging method called Model Based Representation, which can be used to reconstruct electric parameters from the model in Equation (4) and present a visualization to the characteristics of the materials.

### 6.1. Absolute

An absolute imaging method, also called static imaging, is used when the reconstruction is made for measurements on one moment in time and no comparison is made between different frequencies. This method is used in [73], where a wideband EITS system is developed using a linear chirp as excitation frequency and the absolute image can be then obtained for each frequency of interest. However, this absolute imaging can be sensitive to forward modeling errors [54], which could be cancelled with difference imaging.

**Figure 8:**
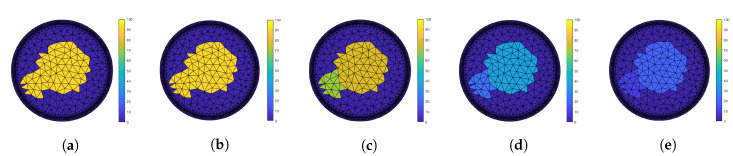
Example of absolute permittivity variation over frequency for an ice and water mixture considering: (**a**) 100 Hz, (**b**) 1 kHz, (**c**) 10 kHz, (**d**) 100 kHz, and (**e**) 1 MHz.

An example of how absolute permittivity varies with frequency is shown in Figure 8 for an ice and water mixture simulation at five different frequencies between 100 Hz and 1 MHz.

### 6.2. Time Difference

Time difference will analyze how the electrical property of interest varies in time, therefore it is also called a dynamic imaging method. This is particularly of interest when analyzing industrial multiphase flows, human blood flow or other processes that quickly change in time.

In this method, the variation $V_{a}$ of an electrical property between time $t_{0}$ and $t_{1}$ for a fixed frequency $f_{0}$ is given by

(22) $$V_{a}\left(EP_{a}\right)=EP_{a}\left(f_{0},t_{1}\right)-EP_{a}\left(f_{0},t_{0}\right)$$

In [74,75], time difference imaging was used in EITS to show the reconstruction results over time for each frequency. While in [74] the frequency variation is discrete, in [75] a binary pseudorandom signal was used so that the frequency measurements can happen simultaneously in time.

### 6.3. Frequency Difference

In some applications where it is not possible or desirable to obtain the material change and variation in time, as for example in medical applications where previous measurements of the patient are not available for comparison, a frequency difference method can be the key for identifying the presence of different substances. This is particularly true for some applications as addressed in [36,76]. Frequency Difference EITS could be a great solution when checking for a stroke which needs to be quickly identified to be treated in ambulances or other locations which do not possess more sophisticated equipment.

#### 6.3.1. Traditional Frequency Difference

In the frequency difference method, the variation $V_{a}$ of an electrical property $EP_{a}$ of a material *a* is

(23) $$V_{a}\left(EP_{a}\right)=EP_{a}\left(f_{i},t_{0}\right)-EP_{a}\left(f_{0},t_{0}\right)$$

where $f_{0}$ is the reference frequency and $f_{i}$ is the frequency value for index *i* at time $t_{0}$. In this static imaging method, the difference between the value of the electrical property of interest in different frequencies is obtained for *a*.

#### 6.3.2. Relative Frequency Difference

In this method, the frequency difference is imaged in relation to another frequency. As explained in [77], the relative variation $RV_{a}$ is

(24) $$RV_{a}\left(EP_{a}\right)=\frac{EP_{a}\left(f_{i},t_{0}\right)-EP_{a}\left(f_{0},t_{0}\right)}{EP_{a}\left(f_{0},t_{0}\right)}$$

and the multi-frequency variation of the contrast α between a material *a* and a material *b* is given by

(25) $$\frac{d\alpha }{\alpha }=RV_{a}\left(EP\right)-RV_{b}\left(EP\right)$$

#### 6.3.3. Weighted Frequency Difference

In case of weighted frequency difference, Equation (23) is changed into

(26) $$V_{a}\left(EP_{a}\right)=\beta \cdot EP_{a}\left(f_{i},t_{0}\right)-EP_{a}\left(f_{0},t_{0}\right)$$

where β is a constant that has a value of 1 in case the frequency difference if not weighted.

Weighted frequency difference is used in [78] to help decrease the effect of boundary interactions.

#### 6.3.4. Adjacent Frequency Difference

Adjacent frequency difference could be weighted or not depending on β and it is defined as

(27) $$V_{a}\left(EP_{a}\right)=\beta \cdot EP_{a}\left(f_{i},t_{0}\right)-EP_{a}\left(f_{i-1},t_{0}\right)$$

where the frequency difference at index *i* is calculated in reference to i−1 instead of a same reference frequency.

### 6.4. Frequency-Time Difference

When both time and frequency variations are taken into consideration for the imaging, then a Frequency-Time Difference method is used. In the literature, Traditional Frequency-Time difference and Relative Frequency-Time Difference were used for EITS applications. However, other variations also considering Weighted Frequency-Time Difference, for example, could also be of interest.

#### 6.4.1. Traditional Frequency-Time Difference

In [79], frequency difference method was also used over time to verify patient evolution after being admitted to the hospital. In this case, two frequency difference images obtained with Equation (23) in time $t_{0}$ and $t_{1}$ can be compared using Equation (22) as

(28) $$V_{a}\left(EP_{a}\right)=\left[EP_{a}\left(f_{1},t_{1}\right)-EP_{a}\left(f_{0},t_{1}\right)\right]-\left[EP_{a}\left(f_{1},t_{0}\right)-EP_{a}\left(f_{0},t_{0}\right)\right]$$

#### 6.4.2. Relative Frequency-Time Difference

In [43], a relative frequency-time imaging method is proposed. In this method, let us assume a variation

(29) $$V_{a}^{0}\left(EP_{a}\right)=EP_{a}\left(f_{1},t_{0}\right)-EP_{a}\left(f_{0},t_{0}\right)$$

(30) $$V_{a}^{1}\left(EP_{a}\right)=EP_{a}\left(f_{2},t_{1}\right)-EP_{a}\left(f_{0},t_{1}\right)$$

When the Relative Variation is of interest, Equations (29) and (30) can be written as

(31) $$V_{a}^{0}\left(EP_{a}\right)=\frac{EP_{a}\left(f_{1},t_{0}\right)-EP_{a}\left(f_{0},t_{0}\right)}{EP_{a}\left(f_{0},t_{0}\right)}$$

(32) $$V_{a}^{1}\left(EP_{a}\right)=\frac{EP_{a}\left(f_{2},t_{1}\right)-EP_{a}\left(f_{0},t_{1}\right)}{EP_{a}\left(f_{0},t_{1}\right)}$$

### 6.5. Material Fraction

The material fraction imaging will represent the percentage of a certain material in the image, which will vary between 0, when the material of interest is not present, and 1, when that element is formed only by that material. This imaging method is very popular in EIT for void fraction representations. In [55,69,70], this imaging method is used for EITS considering all frequency data to generate the material fraction image. In [53] this same imaging method is used in EITS for ice and water fraction representation.

### 6.6. Model Based Representation

This model is not found in the literature so far, however it is our suggestion of a new representation method which we find very promising. We could find some similar investigations in [22,80] done in the nineties, where a parameter based imaging method is used referencing to the low-frequency impedance, high-frequency impedance and capacitive component.

Recently, in [19], it was shown that the relaxation time could be used to identify breast cancer in tissue. Therefore, an image representation of such an electrical property could be used in such an application. Once the frequency behavior of each pixel element in the image is identified, properties such as relaxation time, static permittivity, high frequency permittivity, static conductivity or high frequency conductivity could be obtained and used for imaging.

### 6.7. 3D Imaging

The methods explained in this section can also be used for 3D imaging instead of 2D and they have already been used recently in the literature.

In [14], measurements are made to perform 3D EITS using frequency difference, showing its advantages over time difference imaging for identifying biological materials with distinct frequency behaviors. Electrodes were distributed in different planes of a sphere and each plane represented one image. Multi-plane imaging is also used in [18] for breast imaging.

Another way of reconstructing 3D EITS images is using only one three-dimensional image instead of many planes, as in [15,16], which can bring a complete view of the object in the region of interest. 3D Imaging has been used in these studies together with absolute, time difference and frequency difference imaging methods.

## 7. Conclusions

EITS consists of a choice of sensing method, which is the hardware and includes the electrode geometry and measurement system, and a software, which will implement the reconstruction algorithm and image representation. The necessities of the application will define what the best solution and best parameters for design are. Not always is the top performance an advantage and other concerns such as computation intensity, overall system size or environmental constraints need to be considered.

Circular electrode geometries are recommended for applications that are interested in observing a section of a volume, such as in the medical field. On the other hand, planar electrode geometries can be of interest in applications where the material being investigated is near the sensor region and when it is not possible to surround the object with electrodes, such as in material accretion on surfaces. However, it can present a greater challenge since only limited information is available.

Reconstruction algorithms vary in computational complexity. Fast, lower complexity algorithms such as non-iterative methods can be useful for embedded applications, yet more computationally complex algorithms, such as iterative methods, can provide more accurate reconstruction.

The choice of an imaging method is of equal importance since it allows the correct visualization of the reconstructed area. Absolute imaging allows the visualization of the electrical property of interest at one frequency at a time without any extra data treatment. Time difference imaging shows the variation over time of the electrical property considering the same frequency, which can be of interest when analysing a process that changes over time or for some medical applications such as those that aim at investigating human breathing. Frequency difference imaging focuses on the variation between two specific frequencies, which can be very useful if the material of interest shows a relaxation effect within that frequency range. Material fraction imaging allows quick visualization of the quantity of one material of interest in the selected region and this can be used, e.g., when one specific material should be identified. Model-based representation imaging shows the main electric parameters to characterize the material based on a known model allowing to represent the entire information provided by EITS in just a few plots.

This review aims to provide a reader with an introduction to key aspects of EITS systems and elaborates a concise literature database for further investigation. The reader is familiarized with the topic such that new concepts in related work on EITS can be followed and extended.

## Figures and Tables

**Table 1:** Comparison between EITS digital systems.

Publication	Year	System	Technology	Source	Frequency Range	Frequency Signal	Drive Pattern/Sensing Strategy	Total of Electrodes	Electrode Geometry	Average SNR
[37]	2001	Sheffield Mk 3.5	DSP	Single	2 kHz to 1.6 MHz	Discrete Sinusoidal (30 frequencies), up to 10 simultaneous frequencies	Adjacent	8	2D Circular	40 dB
[14]	2003	UCLH Mk 2	DSP	Single	20 Hz to 1 MHz	Discrete Sinusoidal (30 frequencies), up to 10 simultaneous frequencies	Opposite	64	3D Circular	40 dB
[36]	2006	UCLH Mk 2.5	DSP	Single	20 Hz to 1 MHz	Discrete Sinusoidal (30 frequencies), up to 10 simultaneous frequencies	Opposite	32	2D Circular	40 dB
[18]	2008	-	DSP/FPGA	Multiple	10 kHz to 10 MHz	Discrete Sinusoidal (20 frequencies)	Adjacent	64	3D Circular	84 dB
[39]	2008	OXBACT-5	FPGA	Multiple	1 kHz to 100 kHz	Discrete Sinusoidal (16 frequencies)	-	64	2D or 3D Circular	-
[42]	2014	KHUMark2.5	DSP/FPGA	Multiple	10 Hz to 500 kHz	Discrete Sinusoidal (9 frequencies), up to 6 simultaneous frequencies	Adjacent or Opposite	16	2D Circular	90 dB
[44]	2015	-	FPGA	Single	100 Hz to 10 MHz	Discrete Sinusoidal (47 frequencies)	Adjacent	32	2D or 3D Circular	90 dB
[46]	2019	SWEIT	FPGA	Single	1 kHz to 1.1 MHz	Continuous Chirp	Adjacent	16	2D Circular	56 dB

**Table 2:** Comparison between EITS reconstruction algorithms.

Algorithm	Type	Classification	Publications
Singular Value Decomposition	Non-iterative	Regularization-Based	[21,42,51]
Newton-Raphson	Iterative	Regularization-Based	[17]
Gauss-Newton	Iterative	Regularization-Based	[43]
D-Bar	Non-iterative	Direct	[52]
Optimal First Order Approximation	Non-iterative	Statistical	[23,53]
Group Sparse Reconstruction Algorithm	Iterative	Regularization-Based	[54]
Reconstruction-Classification	Non-iterative	Machine Learning	[55]

## Appendix A. List of Symbols

In Table A1, the main symbols used in this paper can be found together with a description and units.

**Table A1:** Variables and units.

Symbol	Description	Unit
E	Electric Field	$\frac{V}{m}$
ϕ	Electric Potential	*V*
J	Current Density	$\frac{A}{m^{-2}}$
σ	Conductivity	$\frac{S}{m}$
γ	Admittivity	$\frac{S}{m}$
ε	Permittivity	$\frac{F}{m}$
P	Polarization	$\frac{A}{m^{-2}}$
χ	Susceptibility	1
ω	Angular Frequency	$\frac{rad}{s}$
$\tau_{0}$	Relaxation Time	*s*
